# Multidimensional Evaluation of Awareness in Prader-Willi Syndrome

**DOI:** 10.3390/jcm10092007

**Published:** 2021-05-07

**Authors:** Jesús Cobo, Ramón Coronas, Esther Pousa, Joan-Carles Oliva, Olga Giménez-Palop, Susanna Esteba-Castillo, Ramon Novell, Diego J. Palao, Assumpta Caixàs

**Affiliations:** 1 Mental Health Department, Corporació Sanitària Parc Taulí, Universitat Autònoma de Barcelona—CIBERSAM, 08208 Sabadell, Spain; RCoronas@tauli.cat (R.C.); DPalao@tauli.cat (D.J.P.); 2 Department of Psychiatry and Forensic Medicine, Universitat Autònoma de Barcelona, 08208 Sabadell, Spain; 3 Institut d’Investigació i Innovació Parc Taulí (I3PT), 08208 Sabadell, Spain; jcoliva@tauli.cat (J.-C.O.); OGIMENEZ@tauli.cat (O.G.-P.); 4 Insight Barcelona Research Group, Societat Catalana de Psiquiatria i Salut Mental, 08017 Barcelona, Spain; EPousa@santpau.cat; 5 Psychiatry Department, Hospital Universitari St. Pau, 08041 Barcelona, Spain; 6 Statistic Unit, Fundació Parc Taulí I3PT, 08208 Sabadell, Spain; 7 Endocrinology and Nutrition Department, Hospital Universitari Parc Taulí, Corporació Sanitària ParcTaulí—Universitat Autònoma de Barcelona, 08208 Sabadell, Spain; 8 Medicine Department, Universitat Autònoma de Barcelona, 08208 Sabadell, Spain; 9 Specialized Service in Mental Health and Intellectual Disability, Institut Assistència Sanitària (IAS), Parc Hospitalari Martí i Julià, Salt, 17190 Girona, Spain; susanna.esteba@ias.cat (S.E.-C.); ramon.novell@ias.cat (R.N.); 10 Neurodevelopment Group, Girona Biomedical Research Institute—IDIBGI, Institut Assistència Sanitària (IAS), Parc Hospitalari Martí i Julià, Salt, 17190 Girona, Spain

**Keywords:** Prader-Willi Syndrome, awareness, insight, obesity, BMI, functionality

## Abstract

There are no studies about insight or awareness of illness in patients with Prader-Willi Syndrome (PWS). The objective of this study was to explore the level of awareness of the disorder, of the need for medication, and of the social consequences of the disease, as well as of its main symptoms in PWS. We also aimed to explore relationships between awareness and sociodemographic and clinical characteristics, and to compare all data with a matched sample of patients with psychosis. Insight was assessed by an Adapted version of the Scale of Unawareness of Mental Disorder in a cross-sectional pilot study at a University Hospital. Thirty-six individuals with PWS (58.3% women) were included. Results showed that PWS patients had a good awareness of the illness and of the effects of medication, in contrast to a lack of awareness of illness’ social consequences. Awareness of obesity/overweight was excellent, as was the awareness of excessive appetite. Awareness of excessive food intake was only mild. Insight correlated with age and functionality, but not with BMI. PWS patients showed a better insight into the illness but a similar awareness of the effects of the medication and of the social consequences of the disease as compared to schizophrenia-spectrum patients. This profile of insight may have relevant clinical implications.

## 1. Introduction

Prader-Willi Syndrome (PWS) is a genetically determined neurodevelopmental disorder [1], which occurs in approximately one in 15,000 births [2]. The critical region has been identified as 15q11-q13, with the majority of PWS cases (approximately 60%) resulting from a micro-deletion in the chromosome of paternal origin. More than one quarter of cases (35%) are due to maternal uniparental disomy (mUPD), in which two copies of the maternal chromosome are present, and a few cases (<5%) are caused by unbalanced translocations or imprinting center defect [2,3,4]. The main clinical characteristics of this syndrome at birth are severe neonatal hypotonia, facial features, and a failure to thrive with poor suck and feeding difficulties. This initial hypophagia is followed by a hyperphagia in early childhood. The most challenging characteristic is hyperphagia with continuous food foraging that leads to early childhood obesity. For this reason and as they get older, they need to be supervised nearly 24 h a day. Other classical characteristics are developmental delay (with learning and behavioral problems), short stature with small hands/feet, and hypogonadism/hypogenitalism due to growth and other hormone deficiencies [5,6].

Cognitive and intellectual deficits range from borderline, to mild, or moderate intellectual disability (ID) [7]. Likewise, they frequently present challenging behaviors of various types and severities, sometimes related to the core symptoms of the disease but sometimes not [8]. Caregivers usually experience high levels of burden, and previous studies detected that the care of a person with PWS could impact their romantic relationships, performance at work, and quality of sleep, and it also could be related to depressed mood or anxiety [9].

Different psychopathological and behavioral problems in different samples of children and adult patients with PWS have been described [10,11,12,13,14,15]. The main psychiatric comorbidities are affective disorders, psychosis, obsessive-compulsive disorder, and autism spectrum disorder. In a recent review, Guinovart et al. [16] detected a great heterogeneity in their manifestations, which warrants the need for a better identification of their frequency and clinical signs.

The phenomenon of lack of insight in some specific mental illnesses, namely psychotic and bipolar disorders or dementia syndromes and head injury, has been described for decades [17]. Insight has been conceptualized as a dynamic and multidimensional phenomenon [18,19], which may change over time and includes different dimensions that may be relatively independent. David [18] defined three dimensions of awareness: (i) awareness of illness, (ii) ability to relabel symptoms as pathological, and (iii) treatment compliance. Similarly, Amador et al. [20] proposed three main dimensions of insight: unawareness of illness, unawareness of the need for medication, and unawareness of the social consequences of the disease. These authors also pointed out the distinction between unawareness and misattribution of particular psychotic symptoms. This fact was based on the observation that patients may recognize some symptoms but not others and that one may acknowledge the signs of the illness but attribute them to causes other than abnormalities in their mental states. In addition, they remarked the distinction between present and past insight in order to stress its dynamic nature [21].

Schizophrenia and related psychoses are characterized by a striking unawareness of illness and symptoms, with a substantial proportion (up to 80%) of affected individuals showing poor or absent insight into their mentally ill condition and their clinical manifestations. Such reduced insight or illness unawareness has a crucial clinical relevance since it is associated to poor treatment compliance [22,23,24,25], greater risk of relapse [26], as well as to other relevant clinical, social, and interpersonal outcomes [21,27,28,29].

In schizophrenia, insight deficits have been associated with a higher psychopathological severity, low treatment adherence, higher relapse rate, inferior functioning in the community, and globally a worst outcome [21]. They have also been related to cognitive impairment [30,31], metacognitive capacities [32,33,34], and gender differences [35,36].

To our knowledge, no previous study has explored insight and its impact on the evolution of PWS. Our group recently adapted the Scale of Unawareness of Mental Disorder (SUMD) to PWS, in order to provide a means of capturing the insight phenomenon in this syndrome.

The aim of this study was to describe insight in a sample of PWS patients and to explore its relationships with sociodemographic and clinical characteristics. A second aim was to compare insight of PWS patients to that of a sample of patients with psychosis (with and without ID) since previous experience in awareness was focused mainly on psychosis, and no comparisons in awareness between a neurodevelopmental and a neuropsychiatric disorder has been addressed so far.

## 2. Materials and Methods

### 2.1. Design and Sample

A cross-sectional pilot study including a sample of 36 Caucasian adult patients with PWS genetically diagnosed attending the Endocrinology Department, in the Reference Unit for rare diseases at Corporació Sanitària Parc Taulí de Sabadell (Barcelona), was conducted. The sample was obtained from the study “END-GH-2017 Growth hormone treatment in adults with Prader-Willi syndrome: effect on muscle tone assessed by functional magnetic resonance imaging (fMRI) and its relationship with muscle strength and body composition” (Ethical Committee approval on 27 June 2017—Code 2017/010).

A control group of patients with schizophrenia-spectrum psychosis matched by age and gender (*n* = 36) and a second control group of patients with schizophrenia-spectrum psychosis with comorbid ID other than PWS (*n* = 10) were used as a second reference. Both groups were obtained from the “S(M)LD—Long Term Mental Health” Study (Ethical Committee approval on 17 March 2006—Code 2006/510).

The reason for the selection of the psychosis age-sex-matched control group was the expertise of the authors in awareness in this neuropsychopathology, and the reason for the selection of the psychosis group with ID was with the intention to avoid ID bias.

### 2.2. Procedure

All subjects and their legal tutors (for those with legal incapacity) were informed of the purpose of the study and voluntarily agreed to participate and signed an informed consent. None of the patients and families refused to participate. All the data were anonymized in order to preserve confidentiality.

The evaluation began with a questionnaire of demographic, medical, and clinical data, including current treatments, followed by an interview by two experienced psychiatrists (JC and RC). Patients with PWS were previously genetically diagnosed. Anthropometric parameters were also measured.

### 2.3. Assessment

Intellectual developmental disorder and psychiatric diagnosis were obtained according to the fifth edition of the Diagnostic and Statistical Manual of Mental Disorders DSM-5 [37].

Insight was assessed by means of an Adapted version of the Scale of Unawareness of Mental Disorder (SUMD) [24,38]. This is a standardized scale on which ratings are made from a direct patient interview, initially designed to evaluate patients with schizophrenia. The original SUMD has three general insight dimensions that explore unawareness of having a mental disorder (SUMD1), unawareness of the effects of medication (SUMD2), and unawareness of the social consequences of the disease (SUMD3), as well as 17 additional items that assess both unawareness and misattribution of each particular symptom of the disease. Scores in each dimension of insight and in unawareness and misattribution of particular symptoms range from 1 to 5, with higher scores indicating worse insight and attribution.

The definitive Adapted PWS version (APW-SUD) of the SUMD included three general insight dimensions that explore (i) unawareness of presenting PWS (APW Scale for the Unawareness of the Disease, APW-SUD1), (ii) unawareness of the effects of psychopharmacological medication used by the patients (as it was needed, APW-SUD2), and (iii) unawareness of the social consequences of PWS (APW-SUD3), which means any effect on their daily life (ordinary or extraordinary) including aspects of social relationship, education, work, or leisure time [39]. Additionally, items that assess both unawareness and misattribution of each particular symptom of the disease were included, when patients showed presence of that particular symptom. Scores in each dimension of insight and in unawareness and misattribution of particular symptoms range from 1 to 5, with higher scores indicating worse insight and attribution.

Moreover, due to the lack of similar previous scales of insight devoted to PWS, we designed a simple Analytical-Visual Insight Scale and an Analytical-Visual Severity Scale. Both scales are Likert-type and score between 0 and 10, where 0 represents no awareness or severity at all, and 10 represents maximum awareness or severity of the illness, respectively.

Symptoms of PWS in the last two weeks were evaluated with an ad hoc questionnaire determining the presence or absence of clinically relevant symptoms. Body Mass Index (BMI) was assessed at the time of evaluation.

Behavioral and emotional current symptoms in the PWS sample were also measured by the Strengths and Difficulties Questionnaire (SDQ) [40,41,42]. The SDQ is a quick and easy-to-perform questionnaire used in the screening of behavioral problems in childhood and adolescence. The use of these questionnaires was decided on the basis of the difficulties to find an optimal and adapted adult questionnaire valid for PWS and other genetical and neurodevelopmental disorders. This test was translated into Spanish, and it is widely used in Spanish epidemiological studies [43]. It comprises five subscales relevant to measuring internalizing and externalizing problems: Emotional Problems; Problems with Peers, Behavioral Problems, Hyperactivity, and Prosocial Behavior. It has been used in different adult samples in the field of disability diseases [44,45]. In our study, the Parents Version was used, and it was fulfilled by the main caregiver.

Functionality was measured by the Global Assessment of Functioning Scale (GAF) [46].

Psychopathology in the Schizophrenia-spectrum psychosis samples was assessed using the Positive and Negative Syndrome Scale for Schizophrenia (PANSS) [47,48]. Mental Disability was diagnosed following DSM-5 criteria [37]. Premorbid Intelligence Quotient (IQ) in the psychosis samples were estimated through the Verbal Subscale of the WAIS Wechsler Adults Intelligence Scale [49,50]. IQ in the PWS sample was obtained with the KBIT-2 test [51].

All the samples were evaluated at the hospital by the same clinicians (JC and RC). IQ of the PWS sample was obtained by SE-C.

### 2.4. Statistical Analysis

Descriptive analysis was carried out using descriptive statistics (percentages, median, and range, when indicated). For the association and the comparative analysis between groups, we used non-parametric statistics (Mann-Whitney U, Spearman rho correlations, the coefficient of intraclass correlation or chi square, when indicated). IBM SPSS version 21 was used for the statistical analysis.

## 3. Results

### 3.1. Description of the Samples

Sample characteristics of PWS and psychotic patients are described in Table 1. Most of patients with PWS lived with their original families, were studied in an adapted primary school, and worked in an adapted occupational workshop. The evaluation took part in the hospital, except in two cases, one at home and another in his residence due to difficulties for patients and families to travel.

The age-sex-matched sample of patients affected by schizophrenia-spectrum psychosis also included 36 patients (58.3% women). Unlike PWS, some lived with their own families and had studied in a non-adapted primary school and were mainly unemployed.

Biometrical variables significantly varied between samples. Patients with PWS showed a greater BMI than the psychosis sample. Severe obesity Class III (BMI ≥ 40) measured by the WHO criteria [52] were present in ten (27.8%) people affected by PWS and only in one (2.8%) of the matched psychosis sample.

A greater percentage of people with PWS showed at least one somatic illness associated (34 cases, 94.4%), compared with the matched psychosis sample (six cases, 16.7%).

Symptoms of PWS in the last two weeks were described in Table 2 and showed a good stability through the time, except for the excessive food intake, obsessive-compulsive behaviors, self-harm/scratching, language difficulties (e.g., receptive or expressive language deficits or articulation deficits), and daytime sleepiness, that changed depending on the time of evaluation.

Up to 15 cases (41.7%) with PWS showed no current psychiatric DSM-5 diagnosis at all, and 14 cases (38.9%) showed a non-specified behavior disorder at evaluation. Only four cases (11.1%) fulfilled current diagnosis of non-specified psychosis, and three cases (8.3%) of non-specified obsessive-compulsive disorder. The sample of people affected by psychosis included mainly people with schizophrenia (15 cases, 41.7%). Onset and duration of the illness are showed in Table 1.

ID results showed mild ID in 55.6% (20 cases) in the PWS sample. Estimated IQ in the psychosis sample showed good results (mean 91.3, sd. 11.79, range 70–115) and in the psychosis + ID group (mean 61.4, sd. 4.03, range 55–65). In some patients with PWS, due to the severity of the disability, it was not possible to explore some aspects of insight, and resulted in missing data for some analyses (Table 2).

Functionality, measured by the GAF, did not show any significant differences between groups (Table 1).

### 3.2. Insight Characteristics in Patients with PWS

Patients with PWS showed different awareness depending on the evaluated aspect. They showed a good awareness of the illness (median of 1.0, range 1 to 5) and of the effect of the psychopharmacological treatment (median of 2.0, range 1 to 5). By contrast, they showed a lack of awareness of social consequences of the illness (median of 5.0, range 1 to 5) (Table 2).

The awareness of three core symptoms of PWS was of interest: awareness of obesity/overweight and awareness of excessive appetite were excellent (median of 1.0, range 1 to 5); awareness of excessive food intake was only medium (median of 3.0, range 1 to 5) (Table 2).

The insight for the rest of the symptoms (not included in the final version of the scale) was highly variable.

The lack of awareness of the illness, the lack of awareness of the effect of the psychopharmacological treatment, and the lack of awareness of social consequences of the illness did not correlate between them.

The lack of awareness of the effect of the psychopharmacological treatment correlated with the lack of awareness of obesity/overweight (rho = 0.533, *p* < 0.05).

There is a positive significant correlation between lack of awareness of three core symptoms of PWS (obesity/overweight, excessive appetite, and excessive food intake) (*p* < 0.01).

Insight measured globally by the Analytical-Visual Insight Scale was on the average (median of 5.0, range between 0 and 10) (Table 1).

Insight measured globally by the Analytical-Visual Insight Scale did not correlate with the lack of awareness of three core symptoms of PWS (obesity/overweight, excessive appetite, and excessive food intake); however, it correlated negatively with the awareness of the illness (rho = −0.571, *p* < 0.01) and with the awareness of the effect of the psychopharmacological treatment (rho = −0.613, *p* < 0.01).

### 3.3. Relationship between Insight and Clinical Characteristics

#### 3.3.1. Gender, Genetic Subtypes, and ID

Neither gender nor genetic subtypes showed any relationship with insight.

Severity of the ID showed significant relationships with awareness. Patients with borderline or mild ID (*n* = 21) had a better awareness than patients with moderate or severe ID (*n* = 15) in the global insight measured by the Analytical-Visual Scale (U = 75.0; *p* = 0.007) and in the awareness of the excessive food intake (U = 94.0; *p* = 0.03).

#### 3.3.2. Body Mass Index (BMI)

No significant correlations were found between BMI and any of the awareness dimensions, including the Analytical-Visual Insight Scale. BMI in our sample was not related to age, sex, genetic subtype, level of ID, comorbid medical or mental illnesses, or psychopharmacological treatment.

Symptom severity as assessed by the Analytical-Visual Severity Scale did not show a significant correlation with BMI. However, the behavioral problem subscale of the SDQ was found to correlate with BMI (Rho = 0.244, *p* < 0.05).

#### 3.3.3. Functionality

Poor insight of the illness assessed by the APW-SUD1 was found to be related to a worse GAF functionality (rho = −0.482, *p* < 0.01).

Better insight assessed by the Analytical-Visual Insight Scale was found positively related with the functionality of the patients measured through the GAF (rho = 0.671, *p* < 0.01).

#### 3.3.4. Age

The lack of awareness of the illness assessed by the APW-SUD1 was directly related to the age of the patient (rho = 0.450, *p* < 0.01).

Insight measured by the Analytical-Visual Insight Scale also correlated negatively with the age of the patient (rho = −0.545, *p* < 0.01).

#### 3.3.5. Behavioral and Emotional Current Symptoms

Behavioral and emotional current symptoms measured by SDQ Problems with Peers Subscale were found to be related to illness unawareness (rho = −0.529, *p* < 0.01)

### 3.4. Comparison of Insight between the PWS and the Schizophrenia-Spectrum Psychosis Groups

Patients with PWS showed a better illness awareness than those with psychosis (APW-SUD1) (U = 295.0; *p* < 0.001) or psychosis + ID (U = 43.0; *p* < 0.001), but no significant differences were found regarding awareness of the effects of the medication (APW-SUD2) or the social consequences of the disease (APW-SUD3) compared to both groups of psychosis (Table 3).

## 4. Discussion

To our knowledge, this is the first study to analyze the phenomenon of insight in a specific genetic neurodevelopmental disorder, such as PWS. Results reveal that patients with PWS show good illness awareness and good awareness of the effects of psychopharmacological treatment, but a striking lack of awareness of the social consequences of the disease. These results are of crucial clinical relevance since PWS patients believe they are capable of independent living when they are actually not, causing important behavioral disturbances and burden of care. Compared to psychotic disorders, people with PWS showed a better illness awareness. Moreover, the degree of insight was positively related to PWS functionality and was not related to BMI.

One explanation why patients with PWS showed a good insight for the illness could be their psychobiography. They start with symptoms and behavioral problems in early childhood, and live most of their lives with parents, following adapted studies and work positions. In most cases, in developed countries, families and medical and social resources protect the patients since early stages at school, usually in a 24 h-controlled environment. Moreover, in our setting, they follow medical visits in a multidisciplinary team every six months and periodical meetings with other patients and families. These facts could help them to develop a better insight of the disease, reinforced not only by the influence of families, caregivers, and medical staff but also by being stakeholders of specific associations of patients affected by the same syndrome. Conversely, some specific cognitive deficits, paternalism, and isolationism due to a 24 h-controlled life could be the cause of the lack of awareness of the social consequences of the disease, because they are prevented from living some experiences on their own. Autonomy would be the main goal to be achieved in PWS, opening the opportunity to improve insight and self-control through specific interventions. The high influence of socio-cultural factors in the “awareness of the social consequences of the disease” dimension of insight has also been repeatedly found in psychosis [27].

In the present study, awareness of the three core PWS symptoms differed: Awareness of obesity/overweight and awareness of excessive appetite were excellent. By contrast, awareness of excessive food intake was only medium. A possible explanation would be that they know they are obese because they are often asked to step on a scale and judged about their weight. They also are aware of their excessive appetite because they think about food all day long. However, they are not aware of their excessive intake because they put forth great efforts not to eat as much as they would like. In a previous study of insight in psychosis of our group [53], levels of unawareness and misattribution of particular symptoms also varied considerably. Symptoms in which patients showed higher unawareness were those implying social cognition (metacognition) problems, such as delusions, unusual appearance, poor social judgment, and unusual eye contact. Similarly, the highest rates on misattribution were found regarding poor social relationships, whereas the lowest were in delusions and thought disorder, indicating that once aware of these symptoms, subjects tend to attribute them correctly to a mental illness [53]. In this sense, lack of insight could be influenced by failures in Theory of Mind and metacognition [32,33]. Psychotic symptoms were not relevant in our PWS sample, but clinically evident failures in metacognition were detected in 66.7% of our PWS sample. Moreover, a recent systematic review of social cognitive dysfunctions in rare diseases associated with psychiatric symptoms, including PWS, showed specific impairments in Theory of Mind and in a different metacognitive task in PWS [54]. These aspects need to be considered in future studies.

Contrary to our expectations, awareness of the disease was not found to be related to BMI in our sample. One reason could be that up to 18.3% of our sample showed normal BMI probably because of the strict dietary programs followed by parents/caregivers at home with a food safety environment or at the school/adapted work places. Periodical controls at the endocrinological department since the early pediatric stages could be another explanation. In addition, most of them were treated with growth hormone in their childhood, with a known effect regulating height, weight, and, consequently, BMI.

In schizophrenia, there are some interesting differences of insight dimensions related to gender [35,36]. As we noted, in our PWS sample, there was no influence of gender in the insight measures. All the same, different previous studies linked genetic subtype with different psychopathological characteristics [14,55], but in our sample, there was no influence of genetic subtype on insight dimensions.

General intellectual functioning has consistently been related to insight in psychosis. According to our results, this would be the same in PWS, since a higher ID was shown to correlate significantly with worse insight, both with the Analytical-Visual Scale and with the awareness of excessive food intake subscale.

Interestingly, the global insight measured by the Analytical-Visual Insight Scale detected the differences better than the specific dimensional evaluations of the clinician with the APD-PWS. In our opinion, this simple Analytical-Visual Scale could be a good introduction to the insight dimension in future studies either in PWS or in non-PWS cohorts with similar characteristics.

The influence of age in our sample is complex. To our surprise, awareness of the illness measured both by the APD-PWS1 and the Analytical-Visual Severity Scale was greater in younger than in older patients with PWS. One possible explanation could be the presence of cognitive difficulties, probably greater in older patients. Another possible reason may be the early implication of younger patients and parents in specific associations and psychoeducational programs, together with the early interventions performed by the medical teams in specialized units that help to acquire illness awareness. Another possible explanation of these awareness age differences could be the implication of stigma: Several younger patients without relevant cognitive limitations could participate in out-family environments (for example, non-adapted educational or non-adapted working environments), but they could be suffering from stigma and discrimination because they do not have the sometimes familial protections of the older.

As expected, a better insight was related to a better global functionality, measured with the GAF in our PWS sample. This is similar to findings in psychosis samples [56], and follows the common-sense view that being aware of one’s condition helps to find better ways of adapting to reality globally. On the other hand, GAF is an objective measure, although it only partially correlates with subjective (or caregiver’s) measures of quality of life and functionality. As an example, in a study in schizophrenia using the PSP scale, patients and clinicians only marginally converge on their judgments concerning the psychosocial functioning of the patients, and patients’ insight may have a moderating role on the approximation of agreement between self- and third-party ratings [57]. Moreover, gender (and other relevant known factors) could also influence these relationships too. In our previous gender analysis in schizophrenia and related disorders, functionality measured by the GAF was also associated in the final model to some dimensions of insight, only in men [35]. Future studies with larger samples are needed to explore the role of gender in the impact of insight in the functionality of patients with PWS, and perhaps using different functionality scales.

Relationships found between the different dimensions of insight and several behavioral and psychopathological factors as measured by the SDQ are of interest. A worse insight of the illness and a worse insight of the effect of the psychopharmacological treatment were related to higher emotional (internalized) symptoms measured by the SDQ Emotional Symptoms Subscale, but the directionality of the influence need to be studied in longer prospective studies. Both issues could be related to stigma aspects or to the self-awareness of the difficulties of the illness. A worse insight of the illness could also be related to more problems with peers.

More behavioral problems with the SDQ subscale were found to be related to a worse insight of the social consequences of the disorder, perhaps due to difficulties in metacognition and mediated by the severity of the illness and/or the severity of the ID.

Excessive appetite was related to higher hyperactivity symptoms and more problems with peers, possibly mediated by the specific food behavior (foraging for food, food stealing and sneaking, insistence in more food, etc.), without self-awareness of the patient. In fact, it is possible that different dimensions of behavioral and emotional symptoms are related to different causes.

In summary, patients with PWS showed different awareness depending on the aspect we evaluated. They showed a good awareness of the illness and of the effect of the psychopharmacological treatment. By contrast, they showed a lack of awareness of the social consequences of the illness. Awareness of three core PWS symptoms also showed different scenarios: Awareness of obesity/overweight was excellent, as well as the awareness of excessive appetite. Awareness of excessive food intake was only medium. Insight in PWS was not related to BMI but correlated with functionality and age.

Patients with PWS showed a better insight in the illness but the same insight in the effects of the medication and in the social consequences of the disease compared to a sample of patients with schizophrenia-spectrum psychosis either with or without ID. Further studies are needed to corroborate our findings and their repercussion in the everyday life of patients with PWS.

## 5. Strengths and Limitations

The main strengths of the analyses are the originality of the approach in a field of research with putative relevant influence in the evaluation of the patients and the possibly future design of behavioral-cognitive strategies of treatment in this difficult-to-treat, genetically mediated neurodevelopmental disease.

The main limitations of the study include the limited sample size (although we included the majority of the population attended in our area). Additionally, due to the preliminary nature of the research, we did not control for multiple comparison bias. Additionally, we did not control for stigma or specific cognitive abilities. As usual in this type of studies, there is a possible tendency toward cognitive bias and socially desirable responses in the results. Another limitation is the lack of comparison with a control group with only ID without psychosis; further studies taking into account all these issues are warranted.

## 6. Conclusions

PWS patients show a good awareness of the illness as well as a good awareness of the effects of medication. However, they show a lack of awareness of the social consequences of the disease that may affect their personal relationships and lead to behavioral problems in their daily life. This profile of awareness is qualitatively different from that in psychosis, and seems to be more influenced by cultural factors such as the protective practices that parents and caregivers exert over people affected by this syndrome in our cultural setting.

## Figures and Tables

**Table 1:** Sociodemographic, clinical, and psychometric description of the samples.

Characteristics	Prader-Willi Syndrome Sample (*n* = 36), S1	PsychosisSample (*n* = 36), S2	Intellectual Disability + Psychosis (*n* = 10), S3	Sample vs. Sample, U, p/Chi, p (df)
Age (years): median (range)	27.7 (18.6–51.2)	27.5 (18.7–50.5)	46.1 (21.5–76.5)	S1 vs. S2, 0.937 S1 vs. S3, **<0.001**
Sex:				
- Female, *n* (%)	21 (58.3)	21 (58.3)	4 (40.0)	S1 vs. S2, 0.157 (1)
- Male, *n* (%)	15 (41.7)	15 (41.7)	6 (60.0)	S1 vs. S3, 0.555 (1)
Residence, *n* (%)				S1 vs. S2, **<0.001** (4) S1 vs. S3, **0.006** (3)
- Parents & original family	31 (86.1)	28 (77.8)	5 (50.0)	
- Institution	5 (13.9)	1 (2.8)	2 (20.0)	
- Own family (couple or siblings)	-	3 (8.3)	2 (20.0)	
- Living alone	-	1 (2.8)	-	
- Homeless	-	3 (8.3)	1 (10.0)	
Educational level, *n* (%)				S1 vs. S2, **<0.001** (5) S1 vs. S3, **0.009** (4)
- Without Literacy	2 (5.6)	1 (2.8))	-	
- Read & Write	11 (30.6)	3 (8.3)	6 (60.0)	
- Non-Adapted Primary school	1 (2.8)	24 (66.7)	3 (30.0)	
- Adapted Primary school	13 (36.1)	-	-	
- Non-Adapted Secondary school	-	8 (22.2)	-	
- Adapted Secondary school	9 (25.2)	-	1 (10.0)	
Employment situation, *n* (%)				S1 vs. S2, **<0.001** (5) S1 vs. S3, **<0.001** (5)
- Active (adapted job)	18 (50.0)	2 (5.6)	1 (10.0)	
- Active (Non-Adapted job)	-	2 (5.6)	-	
- Unemployed	15 (41.7)	13 (36.1)	3 (30.0)	
- Permanent handicapped	1 (2.8)	12 (33.3)	6 (60.0)	
- Student	1 (2.8)	4 (11.1)	-	
- Housework	1 (2.8)	3 (8.3)	-	
Weight (kg): median (range)	85.5 (46.0–140.0)	73.2 (45.4–168.0)	78.4 (54.5–87.5)	S1 vs. S2, **0.005** S1 vs. S3, 0.111
Height (m): median (range)	1.55 (1.41–1.90)	1.65 (1.53–1.90)	1.7 (1.60–1.78)	S1 vs. S2, **<0.001** S1 vs. S3, **0.002**
BMI: median (range)	33.7 (20.4–64.7)	27.3 (16.1–53.0)	24.7 (21.0–33.1)	S1 vs. S2, **<0.001** S1 vs. S3, **0.002**
BMI following WHO Classification, *n* (%)				S1 vs. S2, **<0.001** (5) S1 vs. S3, **0.008** (4)
- Under normality (<18.5)	-	2 (5.6)	-	
- Normal (18.5–24.99)	3 (8.3)	6 (16.7)	4 (40.0)	
- Overweight (25–29.99)	4 (11.1)	21 (58.3)	4 (40.0)	
- Mild obesity—Obesity Class I (30–34.99)	15 (41.7)	6 (16.7)	2 (20.0)	
- Medium obesity—Class II (35–39.99)	4 (11.1)	-	-	
- Severe obesity—Class III (>= 40)	10 (27.8)	1 (2.8)	-	
Genetical characteristics, *n* (%)		-	-	-
- Microdeletions	25 (69.4)			
- Disomy, mutations or imprinting	9 (25.0)			
- Unavailable	2 (5.6)			
Associated relevant physical disease, *n* (%)				S1 vs. S2, **<0.001** S1 vs. S3, 0.201
- Yes	34 (94.4)	6 (16.7)	8 (80.0)	
- No	2 (5.6)	30 (83.3)	2 (20.0)	
Current psychiatric DSM 5 Diagnosis, *n* (%)				-
- None	15 (41.7)	-	-	
- Behaviour Disorder Non-Specified	14 (38.9)	-	-	
- Non-Specified Psychosis	4 (11.1)	9 (25.0)	5 (50.0)	
- Non-Specified O-CD	3 (8.3)	-	-	
- Schizophrenia	-	15 (41.7)	2 (20.0)	
- Schizoaffective Disorder	-	8 (22.2)	2 (20.0)	
- Delusional Disorder	-	-	1 (10.0)	
Psychopharmacological Treatment, *n* (%)				-
- Any of the following drugs	26 (72.2)	36 (100.0)	10 (100.0)	
- Antipsychotics	10 (27.6)	36 (100.0)	10 (100.0)	
- Benzodiazepines	6 (16.7)	23 (69.3)	7 (70.0)	
- Antidepressants	18 (50.0)	17 (47.2)	3 (30.0)	
- Topiramate	12 (33.3)	1 (2.8)	-	
- Zonisamide	5 (13.9)	2 (5.6)	-	
Onset of the illness (Psychosis samples), median (s.d.), range	-	21.3 (6.90) (10.0–45.6)	32.8 (13.0–60.5)	S2 vs. S3, **0.008**
Duration of the illness (Psychosis samples), median (s.d.), range	-	7.7 (7.71) (0.3–31.4)	7.4 (0.3–52.5)	S2 vs. S3, 0.298
DSM 5 Intellectual Disability, *n* (%)				-
- No	-	36 (100.0)	-	
- Borderline with normality	1 (2.8)	-	-	
- Mild Intellectual Disability	20 (55.6)	-	10 (100.0)	
- Moderate Intellectual Disability	13 (36.1)	-	-	
- Severe Intellectual Disability	2 (5.6)	-	-	
IQ KBIT-2 (PWS sample) or Estimated IQ (Psychosis samples): median (range)	65.0 (40–99)	90.0 (70–115)	62.0 (55–68)	S1 vs. S2, **<0.001** S1 vs. S3, 0.466
GAF median (s.d.), range	65.0 (10.0–80.0)	65.0 (35.0–80.0)	52.5 (45.0–75.0)	S1 vs. S2, 0.630 S1 vs. S3, 0.096
AV Scale of Global Severity of Prader-Willi Syndrome: median (range)	8.0 (2–10)	-	-	-
AV Scale of Global Awareness of Prader-Willi Syndrome: median (range)	5.0 (0–10)	-	-	-
SDQ Emotional Symptoms Subscale ^a^: median (range)	3.0 (0–8)	-	-	-
SDQ Behavioural Problems Subscale ^b^: median (range)	5.0 (0–8)	-	-	-
SDQ Hyperactivity Subscale ^c^: median (range)	4.0 (1–6)	-	-	-
SDQ Problems with Peers Subscale ^d^: median (range)	6.0 (0–10)	-	-	-
SDQ Prosocial Behaviours Subscale ^e^: median (range)	8.0 (1–10)	-	-	-
SDQ Total. median (range) ^f^	18.0 (7–25)	-	-	-
PANSS Total: median (range)	-	83.5 (46–125)	93.0 (25.0–121.0)	S2 vs. S3, 0.230
PANSS Positive: median (range)	-	20.0 (10–33)	17.0 (11.0–19.0)	S2 vs. S3, 0.113
PANSS Negative: median (range)	-	19.5 (10–42)	31.5 (5.0–43.0)	S2 vs. S3, 0.064
PANSS General: median (range)	-	42.0 (23–64)	47.5 (5.0–65.0)	S2 vs. S3, 0.407

U: U of Man-Whitney. Chi: Chi Square Test. *p*: signification. df: degree of freedom. BMI: Body Mass Index. WHO: World Health Organization. GAF: Global Assessment of Functioning. O-C: Obsessive-Compulsive Disorder. IQ: Intelligence quotient. SDQ: Strengths and Difficulties Questionnaire. PANSS: Positive and Negative Syndrome Scale for Schizophrenia. AV: Analytical-Visual. ^a^ SDQ Emotional Symptoms Subscale Interpretation: Normal (0–3), Borderline (4), Out of the norm (5–10). ^b^ SDQ Behavioral Problems Subscale Interpretation: Normal (0–2), Borderline (3), Out of the norm (4–10). ^c^ SDQ Hyperactivity Subscale Interpretation: Normal (0–5), Borderline (6), Out of the norm (7–10). ^d^ SDQ Problems with Peers Subscale Interpretation: Normal (0–2), Borderline (3), Out of the norm (4–10). ^e^ SDQ Prosocial Behaviors Subscale: Normal (6–10), Borderline (5), Out of the norm (0–4). ^f^ SDQ Total Interpretation: Normal (0–13), Borderline (14–16), Out of the norm (17–40). PANSS: Positive and Negative Syndrome Scale. Significant associations in bold.

**Table 2:** Symptomatic profile and Awareness following the APW-SUD (Prader-Willi Syndrome sample).

Characteristics	Clinician’s Prader-Willi Syndrome Sample Evaluation (*n* = 36)
Item 1: Presence of the Illness	
- Yes, *n* (%)	36 (100.0)
Item 1 patients evaluated: *n* (%)	32 (88.9)
Item 1 score: Awareness of the illness, median (range)	1.0 (1–5)
Item 2: Presence of Psychopharmacological treatment *	
- Yes, *n* (%)	26 (72.2)
Item 2 patients evaluated: *n* (%)	22 (61.1)
Item 2 score: Awareness of the effect of the Psychopharmacological treatment *, median (range)	2.0 (1–5)
Item 3: Presence of Social Consequences of the illness	
- Yes, *n* (%)	36 (100.0)
Item 3 patients evaluated: *n* (%)	30 (83.3)
Item 3 score: Awareness of Social Consequences of the illness, median (range)	5.0 (1–5)
Item 4: Presence of Obesity/Overweight	
- Yes, *n* (%)	33 (91.7)
Item 4 patients evaluated: *n* (%)	29 (80.6)
Item 4 score: Awareness of Obesity/Overweight **, median (range)	1.0 (1–5)
Item 5: Presence of Excessive appetite	
- Yes, *n* (%)	34 (94.4)
Item 5 patients evaluated: *n* (%)	31 (86.1)
Item 5 score: Awareness of Excessive appetite **, median (range)	1.0 (1–5)
Item 6: Presence of Excessive food intake	
- Yes, *n* (%)	28 (77.8)
Item 6 patients evaluated: *n* (%)	27 (75.0)
Item 6 score: Awareness of Excessive food intake **, median (range)	3.0 (1–5)
Item 7: Presence of Obsessive-Compulsive Behaviours	
- Yes, *n* (%)	19 (52.8)
Item 7 patients evaluated: *n* (%)	16 (44.4)
Item 7: Awareness of Obsessive-Compulsive Behaviours **, median (range)	1.0 (1–5)
Item 8: Presence of Hyperactivity	
- Yes, *n* (%)	4 (11.1)
Item 8 patients evaluated: *n* (%)	33 (91.7)
Item 8: Awareness of Hyperactivity **, median (range)	5.0 (5–5)
Item 9: Presence of Self harm/Scratching/Onicophagia	
- Yes, *n* (%)	27 (75.0)
Item 9 patients evaluated: *n* (%)	25 (69.4)
Item 9: Awareness of Self harm/Scratching/Onicophagia **, median (range)	2.0 (1–5)
Item 10: Presence of Heteroaggressivity	
- Yes, *n* (%)	9 (25.0)
Item 10 patients evaluated: *n* (%)	8 (22.2)
Item 10: Awareness of Heteroaggressivity **, median (range)	3.0 (1–5)
Item 11: Presence of Clinically metacognitive impairment	
- Yes, *n* (%)	24 (66.7)
Item 11 patients evaluated: *n* (%)	21 (58.3)
Item 11: Awareness of Clinically metacognitive impairment **, median (range)	3.0 (1–5)
Item 12: Presence of Language difficulties	
- Yes, *n* (%)	11 (30.6)
Item 12 patients evaluated: *n* (%)	9 (25.5)
Item 12: Awareness of Language difficulties **, median (range)	5.0 (1–5)
Item 13: Presence of Corporal/Morphological alterations	
- Yes, *n* (%)	7 (19.4)
Item 13 patients evaluated: *n* (%)	5 (13.9)
Item 13: Awareness of Corporal/Morphological alterations **, median (range)	1.0 (1–1)
Item 14: Presence of Daytime sleepiness	
- Yes, *n* (%)	23 (63.9)
Item 14 patients evaluated: *n* (%)	21 (58.3)
Item 14: Awareness of Daytime sleepiness **, median (range)	4.0 (1–5)
Item 15: Presence of Psychological rigidity/Obstinacy	
- Yes, *n* (%)	24 (66.7)
Item 15 patients evaluated: *n* (%)	24 (66.7)
Item 15: Awareness of Psychological rigidity/Obstinacy **, median (range)	4.0 (1–5)
Item 16: Presence of Sensory-perceptive alterations	
- Yes, *n* (%)	8 (22.2)
Item 16 patients evaluated: *n* (%)	8 (22.2)
Item 16: Awareness of Sensory-perceptive alterations **, median (range)	1.0 (1–5)
Item 17: Presence of Anxiety/Depression	
- Yes, *n* (%)	3 (8.3)
Item 17 patients evaluated: *n* (%)	3 (8.3)
Item 17: Awareness of Anxiety/Depression **, median (range)	1.0 (1–3)

APW-SUD: Adapted Prader-Willi Scale for the Unawareness of the Disorder. Lower values of the APW-SUD represent better awareness (and 1.0 the total awareness); higher values represent worst awareness, with the 5.0 as total lack of awareness. ICC: Intraclass Correlation Coefficient. Chi (df): Chi square test (degrees of freedom). * Awareness of the effect of the psychopharmacological treatment is only evaluated if the treatment is present at the moment of the evaluation. ** Awareness of any individual symptom is only evaluated if the symptom is present at the moment of evaluation or if there is an adequate comprehension of the questions.

**Table 3:** Comparative characteristics of the samples following the adapted APW-SUD (Prader-Willi sample) or validated SUMD (psychosis samples).

Characteristics	Clinician’s Prader-Willi Syndrome Sample Evaluation (*n* = 36), PW1	Clinician’s Psychosis Sample Evaluation (*n* = 36), PS1	Clinician’s Intellectual Disability + Psychosis Sample Evaluation (*n* = 10), PS2	Sample vs. Sample, PW1 vs. PS1: U (*p* Value) PW1 vs. PS2: U (*p* Value)
Item 1: Presence of the Illness				
- Yes, *n* (%)	36 (100.0)	36 (100.0)	10 (100.0)	-
- No, *n* (%)	0 (0.0)	0 (0.0)	0 (0.0)	
Item 1 score: Awareness of the illness, median (range)				PW1 vs. PS1, 295.0 **(>0.001**)
	1.0 (1–5)	3.0 (1–5)	5.0 (3–5)	PS1 vs. PS2, 43.0 **(>0.001**)
Item 2: Presence of Psychopharmacological treatment				
- Yes, *n* (%)	26 (72.2)	36 (100.0)	10 (100.0)	-
- No, *n* (%)	10 (27.8)	0 (0.0)	0 (0.0)	
Item 2 score: Awareness of the effect of the Psychopharmacological treatment *, median (range)				PW1 vs. PS1, 366.0 (0.611)
	2.0 (1–5)	3.0 (1–5)	4.5 (1–5)	PS1 vs. PS2, 75.5 (0.163)
Item 3: Presence of Social Consequences of the illness				
- Yes, *n* (%)	36 (100.0)	36 (100.0)	10 (100.0)	-
- No, *n* (%)	0 (0.0)	0 (0.0)	0 (0.0)	
Item 3 score: Awareness of Social Consequences of the illness, median (range)				PW1 vs. PS1, 469.5 (0.270)
	5.0 (1–5)	5.0 (1–5)	5.0 (2–5)	PS1 vs. PS2, 131.5 (0.569)

APW-SUD: Adapted Prader-Willi Scale for the Unawareness of de Disorder. SUMD: Scales for the Unawareness of Mental Disorder. Lower values in the APW-SUD and the SUMD represent better awareness (and 1.0 the total awareness); higher values represent worst awareness, with the 5.0 as total lack of awareness. ICC: Intraclass Correlation Coefficient (CS1 vs. PW1, CS2 vs. PW2). U: Mann-Whitney U (PW1 vs. PS1, PW1 vs. PS2). * Awareness of the effect of the psychopharmacological treatment in PWS sample was only evaluated if the treatment was present at the moment of the evaluation or if there was an adequate comprehension of the questions (*n* = 22). Significant associations in bold.

## References

[1] Whittington J.E., Holland A.J., Webb T., Butler J., Clarke D., Boer H. (2001). Population prevalence and estimated birth incidence and mortality rate for people with Prader-Willi syndrome in one UK Health Region. J. Med. Genet..

[2] Butler M.G., Miller J.L., Forster J.L. (2019). Prader-Willi Syndrome—Clinical Genetics, Diagnosis and Treatment Approaches: An Update. Curr. Pediatr. Rev..

[3] Bittel D.C., Butler M.G. (2005). Prader–Willi syndrome: Clinical genetics, cytogenetics and molecular biology. Expert Rev. Mol. Med..

[4] Angulo M.A., Butler M.G., Cataletto M.E. (2015). Prader-Willi syndrome: A review of clinical, genetic, and endocrine findings. J. Endocrinol. Investig..

[5] Butler M.G., Manzardo A.M., Forster J.L. (2016). Prader-Willi Syndrome: Clinical Genetics and Diagnostic Aspects with Treatment Approaches. Curr. Pediatr. Rev..

[6] Gil E.M., Giménez-Palop O., Caixàs A. (2018). Tratamiento con hormona de crecimiento en el síndrome de Prader-Willi. Endocrinol. Diabetes Nutr..

[7] Whittington J.E., Holland A.J. (2017). Cognition in people with Prader-Willi syndrome: Insights into genetic influences on cognitive and social development. Neurosci. Biobehav. Rev..

[8] Yang L., Zhan G.-D., Ding J.-J., Wang H.-J., Ma D., Huang G.-Y., Zhou W.-H. (2013). Psychiatric Illness and Intellectual Disability in the Prader–Willi Syndrome with Different Molecular Defects—A Meta Analysis. PLoS ONE.

[9] Kayadjanian N., Schwartz L., Farrar E., Comtois K.A., Strong T.V. (2018). High levels of caregiver burden in Prader-Willi syndrome. PLoS ONE.

[10] Raga L.R. (2003). Fenotipos conductuales en el síndrome de Prader-Willi. Revista Neurol..

[11] Feighan S.-M., Hughes M., Maunder K., Roche E., Gallagher L. (2019). A profile of mental health and behaviour in Prader-Willi syndrome. J. Intellect. Disabil. Res..

[12] Holland A.J., Aman L.C., Whittington J.E. (2019). Defining Mental and Behavioural Disorders in Genetically Determined Neurodevelopmental Syndromes with Particular Reference to Prader-Willi Syndrome. Genes.

[13] Royston R., Oliver C., Howlin P., Dosse A., Armitage P., Moss J., Waite J. (2019). The Profiles and Correlates of Psychopathology in Adolescents and Adults with Williams, Fragile X and Prader–Willi Syndromes. J. Autism Dev. Disord..

[14] Dykens E.M., Roof E. (2008). Behavior in Prader-Willi syndrome: Relationship to genetic subtypes and age. J. Child Psychol. Psychiatry.

[15] Novell-Alsina R., Esteba-Castillo S., Caixàs A., Gabau E., Giménez-Palop O., Pujol J., Deus J., Torrents-Rodas D. (2019). Compulsions in Prader-Willi syndrome: Occurrence and severity as a function of genetic subtype. Actas Esp. Psiquiatr..

[16] Guinovart M., Coronas R., Caixàs A. (2019). Alteraciones psicopatológicas en el síndrome de Prader-Willi. Endocrinol. Diabetes Nutr..

[17] David A.S., Bedford N., Wiffen B., Gilleen J. (2012). Failures of metacognition and lack of insight in neuropsychiatric disorders. Philos. Trans. R. Soc. B Biol. Sci..

[18] David A.S. (1990). Insight and Psychosis. Br. J. Psychiatry.

[19] Marková I., Berrios G. (1995). Insight in clinical psychiatry revisited. Compr. Psychiatry.

[20] Amador X.F., Flaum M., Andreasen N.C., Strauss D.H., Yale S.A., Clark S.C., Gorman J.M. (1994). Awareness of Illness in Schizophrenia and Schizoaffective and Mood Disorders. Arch. Gen. Psychiatry.

[21] Amador X.F., David A.S. (2004). Insight and Psychosis: Awareness of Illness in Schizophrenia and Related Disorders.

[22] Kemp R., David A. (1996). Psychological Predictors of Insight and Compliance in Psychotic Patients. Br. J. Psychiatry.

[23] Sanz M., Constable G., Lopez-Ibor I., Kemp R., David A.S. (1998). A comparative study of insight scales and their relationship to psychopathological and clinical variables. Psychol. Med..

[24] Amador X.F., Strauss D.H. (1993). Poor insight in schizophrenia. Psychiatr. Q..

[25] Bartkó G., Herczeg I., Zádor G. (1988). Clinical symptomatology and drug compliance in schizophrenic patients. Acta Psychiatr. Scand..

[26] David A., Van Os J., Jones P., Harvey I., Foerster A., Fahy T. (1995). TInsight and psychotic illness. Cross-sectional and longitudinal associations. Br. J. Psychiatry.

[27] Lincoln T.M., Lüllmann E., Rief W. (2006). Correlates and Long-Term Consequences of Poor Insight in Patients with Schizophrenia. A Systematic Review. Schizophr. Bull..

[28] Erickson M., Jaafari N., Lysaker P. (2011). Insight and negative symptoms as predictors of functioning in a work setting in patients with schizophrenia. Psychiatry Res..

[29] Massons C., Lopez-Morinigo J.-D., Pousa E., Ruiz A., Ochoa S., Usall J., Nieto L., Cobo J., David A.S., Dutta R. (2017). Insight and suicidality in psychosis: A cross-sectional study. Psychiatry Res..

[30] Nieto L., Cobo J., Pousa E., Blas-Navarro J., Garcia-Pares G., Palao D., Obiols J.E. (2012). Insight, symptomatic dimensions, and cognition in patients with acute-phase psychosis. Compr. Psychiatry.

[31] Aleman A., Agrawal N., Morgan K.D., David A.S. (2006). Insight in psychosis and neuropsychological function. Br. J. Psychiatry.

[32] Pousa E., Duñó R., Navarro J.B., Ruiz A.I., Obiols J.E., David A.S. (2008). Exploratory study of the association between insight and Theory of Mind (ToM) in stable schizophrenia patients. Cogn. Neuropsychiatry.

[33] Pousa E., Duñó R., Brébion G., David A.S., Ruiz A.I., Obiols J.E. (2008). Theory of mind deficits in chronic schizophrenia: Evidence for state dependence. Psychiatry Res..

[34] De Jong S., Van Donkersgoed R.J.M., Arends J., Lysaker P.H., Wunderink L., Van Der Gaag M., Aleman A., Pijnenborg G.H.M. (2016). Metacognition in psychotic disorders: From concepts to intervention. Tijdschr. Psychiatr..

[35] Cobo J., Nieto L., Ochoa S., Pousa E., Usall J., Baños I., González B., Ruiz I., Ruizc A.I. (2016). Insight and gender in schizophrenia and other psychoses. Psychiatry Res..

[36] Cobo J., Labad J., Pousa E., Nieto L., Ochoa S., Usall J., García-Ribera C., Baños I., González B., Massons C. (2020). Exploring the relationship of insight with psychopathology and gender in individuals with schizophrenia spectrum disorders with structural equation modelling. Arch. Women’s Ment. Health.

[37] American Psychiatric Association (2014). Manual Diagnóstico y Estadístico de los Trastornos Mentales [DSM-5].

[38] Ruiz A., Pousa E., Duñó R., Crosas J., Cuppa S., García C. (2008). Spanish adaptation of the Scale to Asses Unawareness of Mental Disorder (SUMD). Actas Esp. Psiquiatr..

[39] Cobo J., Coronas R., Pousa E., Oliva J.C., Giménez-Palop O., Caixàs A. (2021). An Adapted scale to evaluate Insight in Prader-Willi Syndrome. Med. Clin..

[40] Goodman R. (1997). The Strengths and Difficulties Questionnaire: A Research Note. J. Child Psychol. Psychiatry.

[41] Vostanis P. (2006). Strengths and Difficulties Questionnaire: Research and clinical applications. Curr. Opin. Psychiatry.

[42] Giannakopoulos G., Tzavara C., Dimitrakaki C., Kolaitis G., Rotsika V., Tountas Y. (2009). The factor structure of the Strengths and Difficulties Questionnaire (SDQ) in Greek adolescents. Ann. Gen. Psychiatry.

[43] Fajardo Bullón F., León del Barco B., Felipe Castaño E., Ribeiro Dos Santos E.J. (2012). Salud mental en el grupo de edad de 4–15 años a partir de los resultados de la Encuesta Nacional de Salud 2006. Rev. Española Salud Pública.

[44] Glenn S., Cunningham C., Nananidou A., Prasher V., Glenholmes P. (2013). Using the strengths and difficulties questionnaire with adults with Down syndrome. Res. Dev. Disabil..

[45] Brann P., Lethbridge M.J., Mildred H. (2018). The young adult Strengths and Difficulties Questionnaire (SDQ) in routine clinical practice. Psychiatry Res..

[46] Endicott J., Spitzer R.L., Fleiss J.L., Cohen J. (1976). The global assessment scale. A procedure for measuring overall severity of psychiatric disturbance. Arch. Gen. Psychiatry.

[47] Kay S.R., Fiszbein A., Opler L.A. (1987). The Positive and Negative Syndrome Scale (PANSS) for Schizophrenia. Schizophr. Bull..

[48] Martín V.P., Zorita M.J.C. (1994). Validation of positive and negative symptom scale (PANSS) in a sample of Spanish schizophrenic patients. Actas Luso-Esp. Neurol. Psiquiatr. Cienc. Afines.

[49] Weschsler D. (1997). Weschsler Adult Intelligence Scale-III.

[50] Miralbell J., Soriano J.J., López-Cancio E., Arenillas J.F., Dorado L., Barrios M., Cáceres C., Alzamora M.T., Torán P., Pera G. (2010). Vascular Risk Factors and Cognitive Performance in Patients 50 to 65 Years-Old. Neurología.

[51] Kaufman A.S., Kaufman N.L. (2004). Kaufman Brief Intelligence Test.

[52] World Health Organization (2020). BMI Classification. http://www.euro.who.int/en/health-topics/disease-prevention/nutrition/a-healthy-lifestyle/body-mass-index-bmi.

[53] Pousa E., Ochoa S., Cobo J., Nieto L., Usall J., González B., Garcia-Ribera C., Solà V.P., Ruiz A.-I., Baños I. (2017). A deeper view of insight in schizophrenia: Insight dimensions, unawareness and misattribution of particular symptoms and its relation with psychopathological factors. Schizophr. Res..

[54] Morel A., Peyroux E., Leleu A., Favre E., Franck N., Demily C. (2018). Overview of Social Cognitive Dysfunctions in Rare Developmental Syndromes with Psychiatric Phenotype. Front. Pediatr..

[55] Manzardo A.M., Weisensel N., Ayala S., Hossain W., Butler M.G. (2018). Prader-Willi syndrome genetic subtypes and clinical neuropsychiatric diagnoses in residential care adults. Clin. Genet..

[56] Erol A., Delibas H., Bora O., Mete L. (2014). The impact of insight on social functioning in patients with schizophrenia. Int. J. Soc. Psychiatry.

[57] Schaub D., Brüne M., Bierhoff H.-W., Juckel G. (2012). Comparison of Self- and Clinician’s Ratings of Personal and Social Performance in Patients with Schizophrenia: The Role of Insight. Psychopathology.

